# Thrombolytic-Related Asymptomatic Hemorrhagic Transformation Does Not Deteriorate Clinical Outcome: Data from TIMS in China

**DOI:** 10.1371/journal.pone.0142381

**Published:** 2015-11-30

**Authors:** Weihua Jia, Xiaoling Liao, Yuesong Pan, Yilong Wang, Tao Cui, Lichun Zhou, Yongjun Wang, The TIMS-CHINA investigators

**Affiliations:** 1 Center of Stroke, Beijing Tiantan Hospital, Capital Medical University, Beijing, China; 2 National Clinical Research Center for Neurological Diseases, Beijing, China; 3 Center of Stroke, Beijing Institute for Brain Disorders, Beijing, China; 4 Beijing Key Laboratory of Translational Medicine for Cerebrovascular Disease, Beijing, China; 5 Department of Neurology, Beijing Chaoyang Hospital, Capital Medical University, Beijing, China; National University of Singapore, SINGAPORE

## Abstract

**Objective:**

It has been unclear whether thrombolytic-related asymptomatic hemorrhagic transformation (AHT) affects the clinical outcome. To answer this question, we examined whether thrombolytic-related AHT affect short-term and long-term clinical outcome.

**Methods:**

All data were collected from the Thrombolysis Implementation and Monitor of Acute Ischemic Stroke in China (TIMS-China) registry. The patients were diagnosed as having AHT group and non- hemorrhagic transformation (HT) group based on clinical and imaging data. The patients with symptomatic hemorrhagic transformation were excluded from this study. Thrombolytic-related AHT was defined according to European-Australasian Acute Stroke Study (ECASS) II criteria. 90-day functional outcome, 7-day National Institutes of Health Stroke Scale (NIHSS) score, 7-day and 90-day mortalities were compared between two groups. Logistic regression analysis was used to evaluate the effects of AHT on a short-term and long-term clinical outcome.

**Results:**

904 of all 1440 patients in TIMS-China registry were enrolled. 89 (9.6%) patients presented with AHT after thrombolysis within 24-36h. These patients with AHT were more likely to be elder age, cardioembolic subtype, and to have higher National Institutes of Health Stroke Scale score before thrombolysis than patients without AHT. No significant difference was found on the odds of 7-day (95% CI:0.692 (0.218–2.195), (P = 0.532) or 90-day mortalities (95% CI:0.548 (0.237–1.268), P = 0.160) and modified Rankin Score(0–1) at 90-day (95% CI:0.798 (0.460–1.386), P = 0.423) or modified Rankin Score(0–2) at 90-day (95% CI:0.732 (0.429–1.253), P = 0.116) or modified Rankin Score(5–6) at 90-day (95% CI:0.375 (0.169–1.830), P = 0.116) between two groups.

**Conclusions:**

Thrombolytic-related AHT does not deteriorate short-term and long-term clinical outcome.

## Introduction

Intravenous thrombolysis with alteplase (recombinant tissue plasminogen activator) is the most effective therapy for acute ischemic stroke [1,2]. However, the potential increased risk of thrombolytic-related hemorrhagic transformation (HT), including Asymptomatic Hemorrhagic Transformation (AHT), limits its application [3–6]. The incidence of AHT after intravenous thrombolysis was found to be 9.9% from combined CT-based data of the NINDS rt-PA trial and the ATLANTIS trial [2,7,8]. Thrombolytic-related symptomatic HT is associated with worse functional outcome. However, it is controversial whether AHT directly leads to negative prognosis. Some studies suggest that prognosis of patients with asymptomatic HT may be innocuous; some studies confirmed that patients with AHT compared with those without HT had a negative effect on functional outcome [9–12]. Therefore, we hypothesized that thrombolytic AHT might be associated with negative neurologic recovery. The aim of this study is to identify risk factors of thrombolytic-related AHT as well as to examine the influence of AHT on short-term and long-term clinical outcome with TIMS-China registry.

## Materials and Methods

### Subjects

All of data were collected from the Thrombolysis Implementation and Monitor of acute ischemic Stroke in China (TIMS-China) registry [13], a national prospective stroke registry of thrombolytic therapy with intravenous alteplase in patients with acute ischemic stroke in China. In nationwide, 67 centers participated in this stroke registry between May 2007 and April 2012. Demographics on clinical characteristics, cranial CT scans, medical therapy and thrombolysis information were collected. All patients were followed up for 3 months.

**Fig 1:**
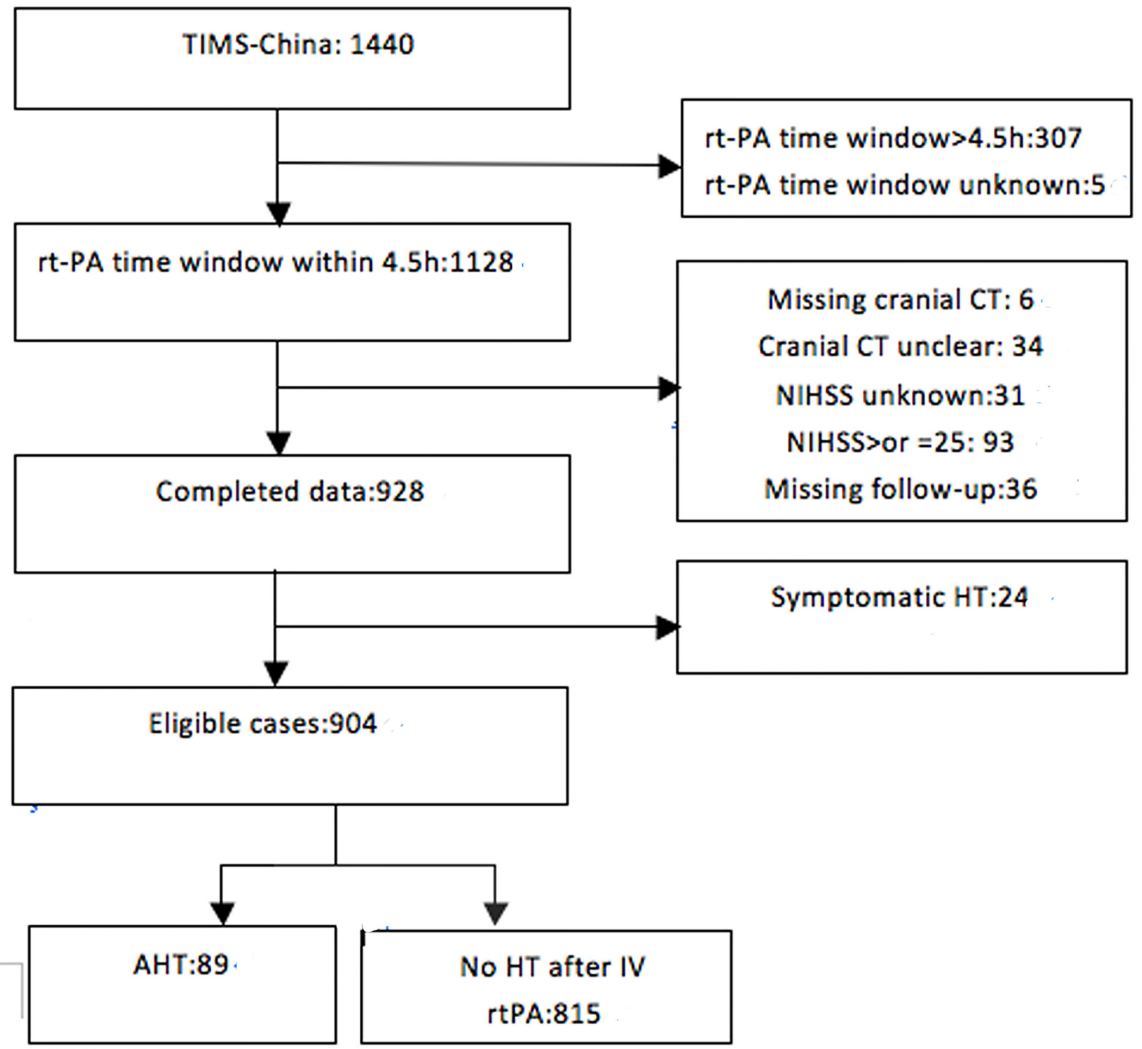
Numbers of eligible patients in TIMS-China.

Eligible patients received intravenous thrombolysis within 4.5-hour. Patients with incomplete data and thrombolysis time window>4.5h or unknown were excluded. Patients with NIHSS score ≥25were excluded. Patients with symptomatic HT were excluded from this study. Eligible patients were divided into two groups: the AHT group, which had asymptomatic hemorrhagic transformation; and the non-HT group, which had no thrombolytic-related hemorrhagic transformation (Fig 1).

### Ethics Statement

The registry of TIMS-China was approved by ethics committee of Beijing Tiantan hospital in 2006. The data from the TIMS-China registry was anonymous before access and analysis by all researchers and patient treatment was assigned and determined independent of all researchers. All patients had written informed consent before being entered in the registry.

### Outcome analysis

Follow-up CT scans for patients were collected at 24–36 hours and 7-day. The diagnosis of HT on brain images was determined independently by 2 neurologists blinded to clinical data. If there was a disagreement between 2 neurologists, a third neurologist was consulted, and a consensus decision was reached. Hemorrhagic transformation must be scored according to the ECASS II classification [2] during all follow-up CT scans: hemorrhagic infarcts (type 1 or 2) or parenchymal hematomas (type 1 or 2). HT was also often divided into symptomatic HT and AHT groups based on the deterioration in neurologic status. AHT, according to ECASS II criteria, was defined as NIHSS score less than 4 points worsening from baseline in neurological status in the first 24 hours after thrombolysis with the presence of hemorrhagic transformation on the follow-up CT scan at 24-36h [2]. The primary outcome measure collected included functional outcome at 90-day (functional independence was defined as a modified Rankin Scale (mRS) score of 0 to 6), NIHSS at 7-day, and mortality at 7-day and 90-day.

### Statistical Analysis

Results were reported as mean ± SD or the frequency of categorical variables. Pearson chi-square (χ2) test was used for comparisons of categorical variables. Continuous variables were analyzed using t test or Mann-Whitney U test. Percentage proportions of outcome events were calculated by dividing the number of events by the total number of patients. For each outcome variable, a separate multivariable logistic regression was performed. Odds ratios (ORs) with its 95% confidence intervals (CI) were calculated by using non-HT group after thrombolysis as the reference group. Two-sided values of P less than .05 were considered statistically significant. All statistical analyses were performed using SAS software version 9.3 (SAS Institute Inc, Cary, NC).

## Results

904 patients (62.8%) were enrolled from a patient population of 1440 from 67 centers across TIMS-China. Our exclusion criteria were as follows: (1) incomplete neuroimaging workups (n = 40), (2) loss to follow-up (n = 36), (3) thrombolysis time window>4.5h or unknown (n = 312), (4) symptomatic HT (n = 24), (5) Patients with severe stroke with a NIHSS score >25(n = 93). It had been reported that 89(9.6%) patients were transformed to AHT in China (Fig 1), 24 (2.5%) patients had symptomatic HT. The characteristics of the patients with AHT group and non-HT group after intravenous thrombolysis are shown in Table 1, which indicates that more patients with the history of atrial fibrillation, high NIHSS score and elderly age are with AHT after thrombolytic therapy and fewer patients with a history of transient ischemic attack are with AHT. Proportion of patients transformed with asymptomatic hemorrhage after thrombolytic therapy were as follow: HT2 subtype highest proportion (38 cases) > followed HT1 subtype (30 cases) > PH1 subtypes (15 cases) > PH2 subtypes (6 cases). NIHSS score of asymptomatic patients with hemorrhagic transformation after thrombolysis (15.56±6.61) was higher than that of the patients without bleeding transformation after thrombolysis (11.96±6.90) (P<0.001).

**Table 1:** Baseline characteristics of patients between AICH and no HT after thrombolysis.

	AICH Group	Non-HT Group	P-value
	(N = 89)	(N = 815)	
Gender(male)	40 (44.94)	311 (38.16)	0.212
age	66.11±9.73	63.37±11.29	0.034
history of hypertion	49 (55.06)	484 (59.39)	0.430
History of diabetes	12 (13.48)	145 (17.79)	0.308
History atria fibrillation	31 (34.83)	143 (17.55)	<0.001
History of smoke	25 (28.09)	291(35.71)	0.153
Post-thrombolytic antiplatelet use	12 (13.48)	120 (14.72)	0.753
Time of rt-PA given(hour)	2.84±0.74	2.81±0.79	0.822
Serum glucose(mmol/L)	7.16±2.39	7.69±2.90	0.097
Systolic blood pressure(mmHg)	147.54±23.42	148.23±20.59	0.813
Dystolic blood pressure(mmHg)	85.11±13.61	86.00±12.49	0.521
History of hyperlipidema	4 (4.49)	55 (6.75)	0.414
TOAST type			
Atherothrombotic	50 (56.18)	430 (53.09)	<0.001
Lacunar	0 (0.00)	90 (11.11)	
Cardioembolic	31 (34.83)	149 (18.40)	
Other/Unknown	8 (8.99)	141 (17.41)	
Dose of rt-PA(mg/kg)	0.86±0.11	0.86±0.10	0.126
NIHSS score before thrombolysis	15.56±6.61	11.96±6.90	<0.001
History of mRS	3/ 89 (3.37)	35 (4.06)	0.976
History of TIA	3(3.37)	85 (10.43)	0.034
History of stroke	15(16.85)	154 (18.90)	0.639
Occlusion of Total interal carotid artery	8 (10.13)	48 (6.55)	0.233
Occlusion of Proximal middle cerebral artery	8 (10.13)	49 (6.68)	0.255

*NIHSS = National Institutes of Health Stroke Scale; TIA = Transient ischemic attach;* HT, hemorrhagic transformation; AICH, asymptomatic intracerebral hemorrhage; mRS, modified Rankin Scale. TOAST = the trial of org 10172 in acute stroke treatment.

**Fig 2:**
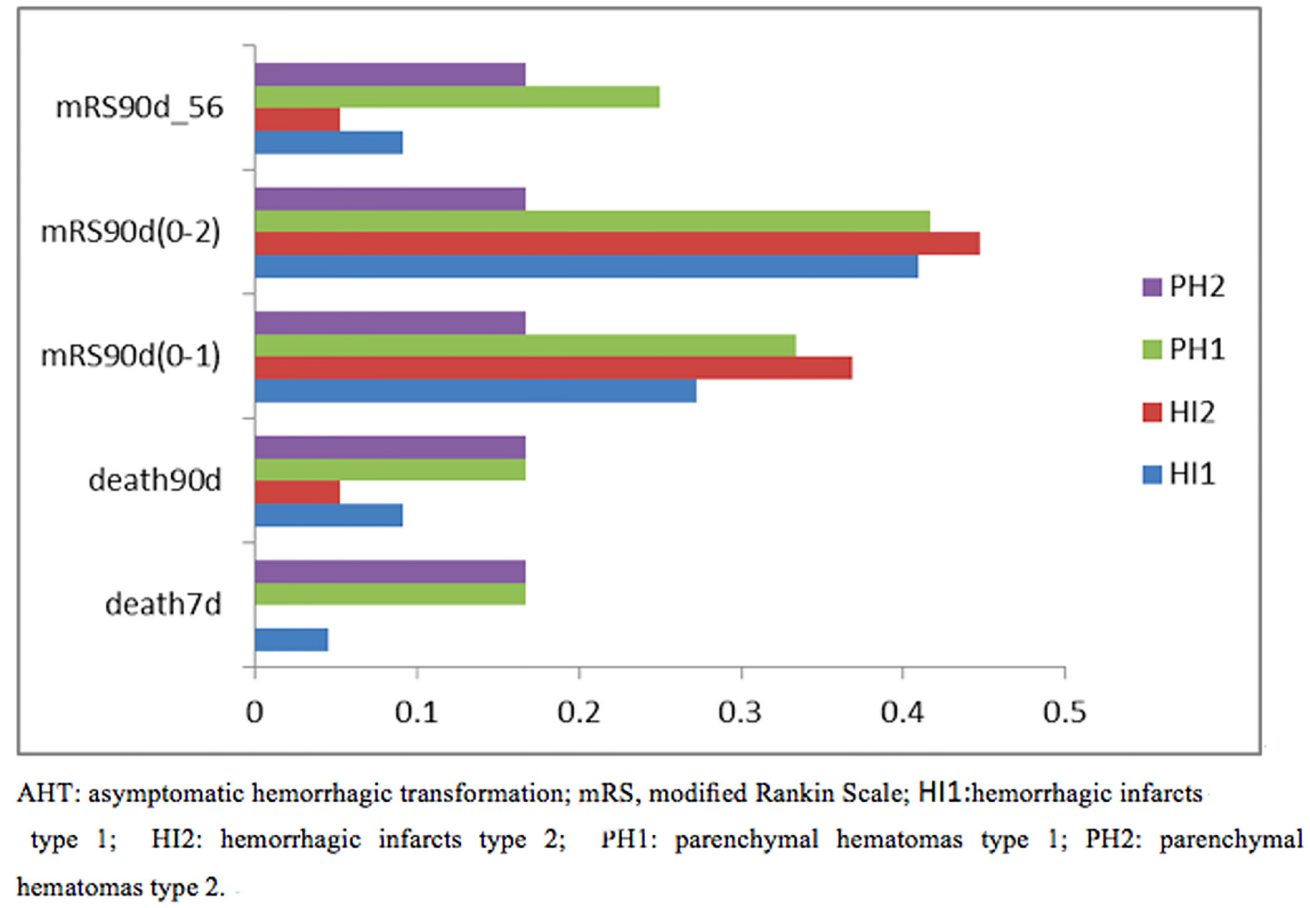
Comparison of function in different subtype of AHT at 7-day and at 90-day in TIMS-china. AHT: asymptomatic hemorrhagic transformation; mRS, modified Rankin Scale; HI1:hemorrhagic infarcts type 1; HI2: hemorrhagic infarcts type 2; PH1: parenchymal hematomas type 1; PH2: parenchymal hematomas type 2.

The proportion of poor outcome in patients with AHT was 13.48% at 3 months after a stroke onset and the patients with non-HT was 11.04%. AHT deteriorated clinical outcomes of 90-day of follow-up (mRS score (0–1 grade) and mRS score (0–2) in patients after thrombolysis when we analyze the data without any adjustment for covariates. After adjustment for baseline relevant factors, no significant difference was found on the odds of 7-day (95% CI:0.692 (0.218–2.195), (P = 0.532) or 90-day mortalities (95% CI:0.548 (0.237–1.268), P = 0.160) and modified Rankin score(0–1) at 90-day (95% CI:0.798 (0.460–1.386), P = 0.423) or modified Rankin score(0–2) at 90-day (95% CI:0.732 (0.429–1.253), P = 0.116) or modified Rankin score(5–6) at 90-day (95% CI:0.375 (0.169–1.830), P = 0.116) between two groups (Table 2). In AHT subgroup analysis, there was a more favorable clinical outcome in HI1and HI2 subgroups than PH1 and PH2 subgroups (Fig 2).

**Table 2:** Functional Outcomes between AHT and non-HT after thrombolysis.

Outcome	AHT	Non-HT,		Unadjusted HR		Adjusted HR	
	(9.9%;)		(90.1%;)	95%CI	P valve	95%CI	P valve
Mortality at 7-day	5 / 89 (5.62)		23 / 815 (2.82)	2.050 (0.760–5.533)	0.1565	0.692 (0.218–2.195)	0.5318
Mortality at 90-day	11 / 89 (12.36)		61 / 798 (7.64)	1.704 (0.861–3.374)	0.1263	0.548 (0.237–1.268)	0.1603
mRS(0–1) at 90-day	27 / 89 (30.34)		403 / 797 (50.56)	0.426 (0.265–0.683)	0.0004	0.798 (0.460–1.386)	0.4231
mRS(0–2) at 90-day	34 / 89 (38.20)		493 / 797 (61.86)	0.381 (0.243–0.598)	0.0001	0.732 (0.429–1.249)	0.2529
mRS(5–6) at 90-day	12 / 89 (13.48)		88 / 797 (11.04)	1.256 (0.657–2.399)	0.4905	0.375 (0.169–1.830)	0.1155

HR, hazard ratios; CI, confidence interval; HT, hemorrhagic transformation; AHT, asymptomatic hemorrhagic transformation; mRS, modified Rankin Scale. Adjusted covariates age, baseline NIHSS, atrial fibrillation, systolic and TOAST criteria using a logistic regression model.

## Discussion

There were remarkably low rates of asymptomatic hemorrhagic transformation (4.5%) [1] in the National Institute of Neurological Disorders and Stroke (NINDS) rtPA Stroke Study. A large observation study of intravenous thrombolysis, the Safe Implementation of Thrombolysis in Stroke-Monitoring Study (SITS-MOST), also showed low rates of asymptomatic hemorrhagic transformation (9.6%) [4]. In TIMS-China, its rate was 9.5%. However, there were higher rates of asymptomatic hemorrhage (39.6%) in the European Cooperative Acute Stroke Study (ECASS-II) [2]. Compared with ECASS-II, the earlier initiation of thrombolysis is the possible reason for the low asymptomatic hemorrhage rates in NINDS, SITS-MOST and TIMS-China. In addition, the direct comparison of the AHT rates among different studies may be hampered by variability in definitions, the earlier initiation of thrombolysis and different image examination time [14–19]. In non-HT group, more patients had TIA history compared with AHT group, which was mainly explained by recurrent TIA increasing collateral circulation. In Asia, many hospitals choose a low-dose intravenous alteplas(IV-tPA) regimens because they believe that racial differences need to reduce dose of treatment in order to decrease rate of hemorrhage transformation. The result of J-MARS showed that 0.6 mg/kg IV-tPA achieved low rates of HT [20]. TIMS-china registry demonstrated the standard-dose IV-tPA therapy was safe for Chinese patients with stroke, without any increased rate of HT or mortality compared with the low-dose Group [21]. In our study, there is no difference of the dose IV-tPA between AHT group and non-HT group.

Previous some studies have shown that thrombolytic-related AHT does not have a negative effect on the short-term or long-term functional outcome. AHT after thrombolysis has a suggestive of vascular recanalization and effective treatment [22]. Moreover, it seems to be a beneficial treatment choice that antiplatelet agents are administered for AHT patients after intravenous thrombolysis [23]. Our novel findings are that among patients who suffered acute ischemic stroke receiving intravenous thrombolysis, the risk of poor outcome for patients with AHT was 0.375-fold at 3 months than for patients without any HT; the risk of mortality for patients with AHT was 0.548-fold at 3 months than patients without any HT. These findings show that clinical outcomes after thrombolysis do not deteriorate in the presence of AHT. Moreover, there is a favorable trend in AHT group, which may be due to improved reperfusion after thrombolysis. Conversely, some studies suggest AHT may not be benign [9,10]. A recent study showed AHT maybe have a negative effect [10]. This differential effect may be due to the AHT populations coming from all ischemic stroke patients, while the AHT patients in our study are from ischemic stroke patients receiving intravenous thrombolysis. Additionally, the use of antiplatelet agents is not prescribed to some patients with AHT in above-mentioned study, which results in increased risk of ischemic stroke one-year recurrence. Our study suggests that elderly, cerebral embolism and higher NIHSS score increase risk of AHT. Similarly, previous studies have linked the factors to increased risk of HT [3,7,8,23,24].

Different from mechanism of symptomatic HT, we speculate that AHT may link with small artery formation rather than large artery formation. It has been showed that thrombolysis after acute ischemic infarction may lead to artery reopening and collateral circulation reperfusion6 [24]. Moreover, the formation of collateral circulation may also improve the functional outcome. There is a favorable trend after thrombolysis-related AHT in our study supports this suggestion.

Our study may have the following limitations. (1) Our sample size was relatively small, and our data exclude severe stroke(NIHSS> or = 25), which maybe have a subtle effect (either positive or negative) of AICH outcome. (2) Another limitation is that the study did not follow the randomized, double-blind principles. Despite these limitations, our results have implications that post-thrombolytic AHT in China does have not a negative effect on clinical prognosis.

## Supporting Information

S1 Table Description and Difference Test of Demographics between Groups (DOCX)

S2 Table Multi-logistic Regression for AHT. (XLS)
