# Supplementary material for: Thrombolytic-Related Asymptomatic Hemorrhagic Transformation Does Not Deteriorate Clinical Outcome: Data from TIMS in China
Source: PLoS One. 2015 Nov 30;10(11):e0142381. doi: 10.1371/journal.pone.0142381 (PMC4664552; doi:10.1371/journal.pone.0142381)
Supplement: S1 Table — (DOCX) [file pone.0142381.s001.docx]

| **--** | | | | | |
| --- | --- | --- | --- | --- | --- |
| **index** | **category** | **Group1 (N=89)** | **Group2 (N=815)** | **statistic** | **P-value** |
| gender | 1 | 49 / 89 ( 55.06) | 504 / 815 ( 61.84) | 1.5549a | 0.2124 |
|  | 2 | 40 / 89 ( 44.94) | 311 / 815 ( 38.16) |  |  |
| age | Mean±Std | 66.11±9.73 | 63.37±11.29 | 2.1237d | 0.0337 |
|  | Median(Q1-Q3) | 68.00(59.00-74.00) | 64.00(56.00-73.00) |  |  |
| h_hypt_01 | 0 | 40 / 89 ( 44.94) | 331 / 815 ( 40.61) | 0.6218a | 0.4304 |
|  | 1 | 49 / 89 ( 55.06) | 484 / 815 ( 59.39) |  |  |
| h_diab_01 | 0 | 77 / 89 ( 86.52) | 670 / 815 ( 82.21) | 1.0378a | 0.3083 |
|  | 1 | 12 / 89 ( 13.48) | 145 / 815 ( 17.79) |  |  |
| h_diab_01 | 0 | 77 / 89 ( 86.52) | 670 / 815 ( 82.21) | 1.0378a | 0.3083 |
|  | 1 | 12 / 89 ( 13.48) | 145 / 815 ( 17.79) |  |  |
| h_afib_01 | 0 | 58 / 89 ( 65.17) | 672 / 815 ( 82.45) | 15.4243a | <.0001 |
|  | 1 | 31 / 89 ( 34.83) | 143 / 815 ( 17.55) |  |  |
| h_smk_6m_01 | 0 | 64 / 89 ( 71.91) | 524 / 815 ( 64.29) | 2.0467a | 0.1525 |
|  | 1 | 25 / 89 ( 28.09) | 291 / 815 ( 35.71) |  |  |
| pmh_ap | 1 | 77 / 89 ( 86.52) | 695 / 815 ( 85.28) | 0.0991a | 0.7530 |
|  | 2 | 12 / 89 ( 13.48) | 120 / 815 ( 14.72) |  |  |
| t7dm_ap | 0 | 56 / 89 ( 62.92) | 117 / 815 ( 14.36) | 122.2950a | <.0001 |
|  | 1 | 33 / 89 ( 37.08) | 698 / 815 ( 85.64) |  |  |
| dhour_tpabegin_stroke | Mean±Std | 2.84±0.74 | 2.81±0.79 | 0.2248d | 0.8222 |
|  | Median(Q1-Q3) | 2.82(2.42-3.32) | 2.83(2.33-3.25) |  |  |
| l_glu | Mean±Std | 7.16±2.39 | 7.69±2.90 | -1.6608d | 0.0968 |
|  | Median(Q1-Q3) | 6.40(5.90-7.50) | 6.90(5.90-8.60) |  |  |
| a_sbp | Mean±Std | 147.54±23.42 | 148.23±20.59 | -0.2366d | 0.8130 |
|  | Median(Q1-Q3) | 150.00(130.00-165.00) | 150.00(134.00-162.00) |  |  |
| a_dbp | Mean±Std | 85.11±13.61 | 86.00±12.49 | -0.6417d | 0.5211 |
|  | Median(Q1-Q3) | 85.00(79.00-92.00) | 87.00(80.00-95.00) |  |  |
| h_lipid_01 | 0 | 85 / 89 ( 95.51) | 760 / 815 ( 93.25) | 0.6683a | 0.4137 |
|  | 1 | 4 / 89 ( 4.49) | 55 / 815 ( 6.75) |  |  |
| is_type_2 | 1 | 50 / 89 ( 56.18) | 430 / 810 ( 53.09) | 24.2814a | <.0001 |
|  | 2 | 0 / 89 (0.00) | 90 / 810 ( 11.11) |  |  |
|  | 3 | 31 / 89 ( 34.83) | 149 / 810 ( 18.40) |  |  |
|  | 4 | 8 / 89 ( 8.99) | 141 / 810 ( 17.41) |  |  |
| dose | Mean±Std | 0.86±0.11 | 0.86±0.10 | 1.5289d | 0.1263 |
|  | Median(Q1-Q3) | 0.90(0.86-0.90) | 0.90(0.86-0.90) |  |  |
| nha | Mean±Std | 15.56±6.61 | 11.96±6.90 | 5.3474d | <.0001 |
|  | Median(Q1-Q3) | 15.00(12.00-20.00) | 11.00(7.00-16.00) |  |  |
| nh7d | Mean±Std | 9.99±8.24 | 5.97±6.90 | 5.1921d | <.0001 |
|  | Median(Q1-Q3) | 10.00(3.00-14.50) | 4.00(1.00-9.00) |  |  |
| h_mrs_01 | 0 | 3 / 89 ( 3.37) | 33 / 813 ( 4.06) | 0.0009b | 0.9763 |
|  | 1 | 86 / 89 ( 96.63) | 780 / 813 ( 95.94) |  |  |
| h_tia_01 | 0 | 86 / 89 ( 96.63) | 730 / 815 ( 89.57) | 4.5498a | 0.0329 |
|  | 1 | 3 / 89 ( 3.37) | 85 / 815 ( 10.43) |  |  |
| h_stroke_01 | 0 | 74 / 89 ( 83.15) | 661 / 815 ( 81.10) | 0.2201a | 0.6390 |
|  | 1 | 15 / 89 ( 16.85) | 154 / 815 ( 18.90) |  |  |
| tica | 0 | 71 / 79 ( 89.87) | 685 / 733 ( 93.45) | 1.4220a | 0.2331 |
|  | 1 | 8 / 79 ( 10.13) | 48 / 733 ( 6.55) |  |  |
| proximalmca | 0 | 71 / 79 ( 89.87) | 684 / 733 ( 93.32) | 1.2942a | 0.2553 |
|  | 1 | 8 / 79 ( 10.13) | 49 / 733 ( 6.68) |  |  |
| distalmca | 0 | 73 / 79 ( 92.41) | 684 / 733 ( 93.32) | 0.0935a | 0.7597 |
|  | 1 | 6 / 79 ( 7.59) | 49 / 733 ( 6.68) |  |  |
| sich_sitsmost | 0 | 88 / 89 ( 98.88) | 815 / 815 (100.00) | c | 0.0985 |
|  | 1 | 1 / 89 ( 1.12) | 0 / &frequencymiss2 (0.00) |  |  |
| sich_ecass2 | 0 | 82 / 89 ( 92.13) | 813 / 815 ( 99.75) | c | <.0001 |
|  | 1 | 7 / 89 ( 7.87) | 2 / 815 ( 0.25) |  |  |
| sich_ninds | 0 | 66 / 89 ( 74.16) | 812 / 815 ( 99.63) | 177.3992b | <.0001 |
|  | 1 | 23 / 89 ( 25.84) | 3 / 815 ( 0.37) |  |  |
| dth_7d | 0 | 84 / 89 ( 94.38) | 792 / 815 ( 97.18) | 1.2620b | 0.2613 |
|  | 1 | 5 / 89 ( 5.62) | 23 / 815 ( 2.82) |  |  |
| dth_90d | 0 | 78 / 89 ( 87.64) | 737 / 798 ( 92.36) | 2.3871a | 0.1223 |
|  | 1 | 11 / 89 ( 12.36) | 61 / 798 ( 7.64) |  |  |
| mrs90d_01 | 0 | 62 / 89 ( 69.66) | 394 / 797 ( 49.44) | 13.1140a | 0.0003 |
|  | 1 | 27 / 89 ( 30.34) | 403 / 797 ( 50.56) |  |  |
| mrs90d_012 | 0 | 55 / 89 ( 61.80) | 304 / 797 ( 38.14) | 18.5871a | <.0001 |
|  | 1 | 34 / 89 ( 38.20) | 493 / 797 ( 61.86) |  |  |
| mrs90d_56 | 0 | 77 / 89 ( 86.52) | 709 / 797 ( 88.96) | 0.4767a | 0.4899 |
|  | 1 | 12 / 89 ( 13.48) | 88 / 797 ( 11.04) |  |  |
